# Altered motility of *Caulobacter Crescentus* in viscous and viscoelastic media

**DOI:** 10.1186/s12866-014-0322-3

**Published:** 2014-12-24

**Authors:** Yukun Gao, Marianna Neubauer, Alexander Yang, Nathan Johnson, Michael Morse, Guanglai Li, Jay X Tang

**Affiliations:** Physics Department, Brown University, Providence, RI 02192 USA

**Keywords:** Bacterial motility, Hydrodynamics, Hyperosmolarity, Rheology, Viscous agent, Viscoelasticity

## Abstract

**Background:**

Motility of flagellated bacteria depends crucially on their organelles such as flagella and pili, as well as physical properties of the external medium, such as viscosity and matrix elasticity. We studied the motility of wild-type and two mutant strains of *Caulobacter crescentus* swarmer cells in two different types of media: a viscous and hyperosmotic glycerol-growth medium mixture and a viscoelastic growth medium, containing polyethylene glycol or polyethylene oxide of different defined sizes.

**Results:**

For all three strains in the medium containing glycerol, we found linear drops in percentage of motile cells and decreases in speed of those that remained motile to be inversely proportional to viscosity. The majority of immobilized cells lost viability, evidenced by their membrane leakage. In the viscoelastic media, we found less loss of motility and attenuated decrease of swimming speed at shear viscosity values comparable to the viscous medium. In both types of media, we found more severe loss in percentage of motile cells of wild-type than the mutants without pili, indicating that the interference of pili with flagellated motility is aggravated by increased viscosity. However, we found no difference in swimming speed among all three strains under all test conditions for the cells that remained motile. Finally, the viscoelastic medium caused no significant change in intervals between flagellar motor switches unless the motor stalled.

**Conclusion:**

Hyperosmotic effect causes loss of motility and cell death. Addition of polymers into the cell medium also causes loss of motility due to increased shear viscosity, but the majority of immobilized bacteria remain viable. Both viscous and viscoelastic media alter the motility of flagellated bacteria without affecting the internal regulation of their motor switching behavior.

**Electronic supplementary material:**

The online version of this article (doi:10.1186/s12866-014-0322-3) contains supplementary material, which is available to authorized users.

## Background

Many gram-negative bacteria depend on appendages such as flagella, pili, and stalks for successful colonization of their environment. It is well known that the mechanical environment can trigger changes in gene expression and biochemical signaling, and subsequently, induce a functional response from the cells [1]. Studies also have shown, however, some aspects of the mechanical regulation are independent of feedback from genetic or biochemical signaling pathways. Examples include the motor’s sensitivity to load in *E. coli* [2,3] and the flicking motion of a uni-flagellated bacterium as it changes swimming direction from backward to forward, caused by a buckling instability in the hook [4,5]. These recent findings motivate testing of other scenarios whereby mechanical properties of the medium directly dictate or alter motility-related functions of a motile bacterium. Such testing requires a convenient, mechanical platform upon which more refined studies may be designed, and through this line of study certain properties of mechanical origin may be sorted out from others due to genetic regulation or biochemical signaling.

Numerous chemical agents have been utilized experimentally to alter the mechanical environment of bacteria, especially impacting their motility. Studies performed decades ago explored the effects of elevated viscosity on the swimming speed of several species of bacteria, using extremely high molecular weight polymers such as methylcellulose and polyvinylpyrrolidone (PVP) [6-8]. It was then recognized that the “gel-like”, or weakly elastic nature of these polymers accounted for enhanced motility of flagellated bacteria [9-12]. Another study shows that for *Vibrio alginolyticus*, the swimming speed of a mutant strain (YM4) expressing only a polar flagellum decreases with increased viscosity in PVP containing media, whereas the strains expressing lateral flagella increase swimming speed with viscosity and attain a peak speed at around 5 cP [13].

From the polymer rheology perspective, the balance between the viscous and elastic effects is highly dependent on the frequency of the mechanical perturbance, which is periodically exerted by the rotating flagella and cell body [14,15]. The so-called frequency-dependent viscoelasticity is generally dictated by polymer size, concentration, and network entanglement [16,17]. It is therefore most informative to characterize the mechanical effects of polymers of well-defined structure and size on bacterial motility. Our choice for this study is the inert polyethylene glycol (PEG) or polyethylene oxide (PEO) of linear structure and with a wide range of sizes available. PEG and PEO are nearly identical polymers, but differentially termed depending on whether the molecular weight is below or above 100,000 daltons.

Mechanical inhibition of flagellar motor rotation, due to elevated medium viscosity or proximity to surfaces, is also known to trigger swarmer-cell differentiation, which is a key step in pathogenesis in some bacterial species [18-21]. Specifically, recent work on *Caulobacter crescentus* shows that jamming of the flagellum rotation caused by pili-mediated surface contact or aggregation of cells induced by polymeric crowding agents triggers a just-in-time secretion of surface adhesins [22]. In light of this finding, we choose *Caulobacter crescentus* as a model species to explore for effects on motility caused by mechanical inhibitions that are distinct in nature and perhaps sensitive to the type and molecular size of viscous agents used.

*Caulobacter crescentus* is a gram-negative, α-proteobacterium often found in nutrient-poor, freshwater environments [23-25]. It has a dimorphic life cycle, spending time both as a non-replicating, motile swarmer cell and as a replicating, sessile, stalked cell. During the swarmer stage of its life, it is uni-flagellated. The rotation of its 5–10 μm long flagellum is entirely responsible for moving its 1–2 μm long crescent-shaped body. The swarmer bacterium eventually matures into a sessile cell, characterized by a long, thin, and rigid cell envelope extension called a stalk [26], which sticks to a nearby surface via an elastic, polysaccharide-based, adhesive holdfast [27-29]. This attachment process is facilitated by their type-IV pili, which extend a few μm from the same pole as the flagellum [30,31], and can retract upon sticking to a surface, or binding of a bacteriophage [32].

**Figure 1:**
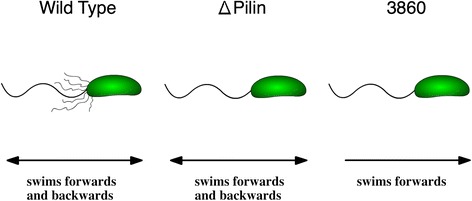
**Schematics of three strains of** ***Caulobacter crescentus*** **used: CB15 wild-type, CB15** Δ**pilin, and SB3860.** The CB15 wild-type swarmer cell expresses pili (thin hairs) and the flagellar motor switches rotation directions to cause alternating forward and backward motion. The Δpilin cell expresses no pili, but the motor operates as the wild-type. The SB3860 (cheR138::Tn5 in YB375) is a double mutant, expressing no pili and moving exclusively forward.

In this study we report on the motility and swimming behavior of *C. crescentus* and its two mutant strains lacking pili in two contrasting media: the Newtonian, i.e., purely viscous, glycerol-water mixture and a viscoelastic medium containing the flexible polymer of PEG or PEO. The wild-type strain CB15 has type IV pili and its flagellar motor switches direction of rotation to cause forward (cell body leading) and backward (cell body trailing behind its flagellum) swimming. CB15 Δpilin is a mutant of the wild-type strain that lacks pili, but it can also swim forward and backward much like the wild-type [33]. SB3860 is a double mutant that lacks pili and can only swim in the forward direction [34,35]. These three strains are illustrated in Figure 1. A close comparison of these three strains allows for determination of specific roles of pili and motor switching, if any, on motility. The goal of our study is to discern to what extent the motion of a uni-flagellated bacterium, with or without pili, is affected by a well-defined, viscoelastic medium, and what features of the motility are changed in comparison with earlier findings on other species of flagellated bacteria [6-8]. The implications of the findings in this study are discussed along with details of the experimental results.

## Results and discussion

Our study was designed to test how motility of uni-flagellated bacteria is affected by certain physical characteristics of the medium. Specifically, we chose polyethylene glycol and polyethylene oxide of selected molecular weights, mixing them into the cell medium in order to examine how changes in polymer size, concentration, and solution viscosity affect bacterial motility. We then compared changes in motility and viability of the three selected strains in a PEG/PYE medium in contrast to cells in glycerol/PYE medium. Finally, we delved into measurements of flagellum motor switching in order to detect subtle changes to its biochemical regulation, if any, caused by the test viscous agent.

### Loss of motility attributable to apparent viscosity of polymer matrix

**Figure 2:**
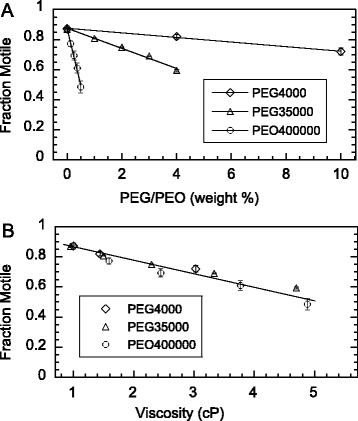
**Loss in motility with polymer added into the medium of synchronized SB3860 swarmer cells. A.** Fraction of motile cells plotted versus PEG 4000, PEG 35000, and PEO 400000 in weight percentage. **B.** The same data collapsed to a single line when re-plotted as a function of viscosity. The errors bars, some smaller than the symbols, represent the standard errors of the mean. The numbers of cells counted were 531 in PEG 4000, 5884 in PEG 35000, and 1191 in PEO 400000.

In a rich growth medium such as peptone yeast extract (PYE), *C. crescentus* usually spends around 30 minutes in the swarmer stage of its life cycle, after which it sheds its flagellum, thereby losing its motility [22]. Mixing PEG or PEO with young swarmer cells, we observed slight decreases in the percentage of motile cells. As a function of the polymer concentration, we found the decrease to be highly sensitive to the polymer size. Whereas 0.5% PEO 400000 caused loss of motility by over 50%, 10% PEG 4000 caused only about 10% loss of motility (Figure 2A). This size sensitivity suggests strongly that rheological property of the polymer matrix might be the main cause for the loss of motility. Indeed, when plotting the same data as a function of the measured shear viscosity (listed on Table 1), we found the same gradual drop in motility, with the combined data in solutions of these three polymers collapsing to a single line (Figure 2B).

**Table 1:** **Measured viscosity of cell media containing listed polymers**

PEG 4000 (%)	1.0	2.0	3.0	4.0	5.0	6.0	7.0	8.0	10.0	
Viscosity (cP)	1.08	1.20	1.44	1.62	1.76	2.06	2.24	2.38	3.03	
PEG 35000 (%)	0.5	1.0	1.5	2.0	2.5	3.0	3.5	4.0	4.5	5.0
Viscosity (cP)	1.24	1.51	1.88	2.32	2.80	3.32	3.90	4.65	5.30	6.34
PEO 400000 (%)	0.125	0.25	0.375	0.5	1.0					
Viscosity (cP)	1.59	2.45	3.77	4.88	14.6					

Measured viscosity versus wt/wt percentage of PEG 4000, PEG 35000 and PEO 400000 daltons. The polymer concentrations were selected to cover the viscosity range up to several times of water. All polymer solutions were made in the Peptone Yeast Extract (PYE) cell medium, the viscosity of which was measured to be 0.98 cP.

### Percentage of motile swarmer cells fell linearly as a function of viscosity

**Figure 3:**
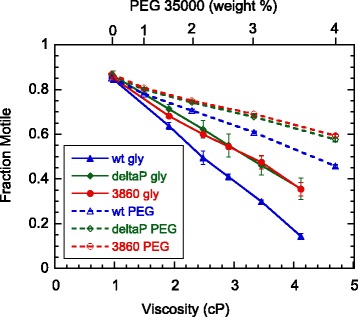
**Motility of swarmer cells as a function of viscosity in viscous and viscoelastic media.** The three types of symbols represent wild-type (wt), Δpilin (deltaP), and SB3860 (3860) strains, in glycerol (solid symbols, with corresponding weight percentages listed on Table 1) and PEG 35000 (empty symbols, with corresponding weight percentages noted on the upper axis). The swarmer cells were counted using videos captured a few minutes after mixing with either pure glycerol or a concentrated PEG solution. The numbers of cells counted were, in PEG 35000, 4639 for wild-type, 4035 for Δpilin, and 5884 for SB3860; and in glycerol, 7174 for wild-type, 3509 for Δpilin, and 3603 for SB3860. The error bars, some smaller than the symbols, are standard errors of three separate sets of measurements.

After being mixed with either a viscoelastic polymer (PEG or PEO) or a viscous medium such as glycerol, loss of motility was observed immediately. The percentage of motile cells decreased nearly linearly with viscosity (Figure 3). As compared with solutions of equivalent viscosity, the glycerol solutions caused a much greater percentage of swarmer cells to become immobile than PEG did. The difference became more apparent in trials performed at higher viscosities. That the trend held across all three strains, CB15 wild-type, CB15 Δpilin, and SB3860 attests to the robustness of the observed property.

Among the three strains, the wild-type cells lost motility more steeply than the other two strains in both types of media as the viscosity increased. This difference suggests a specific role of pili on the loss of motility, which might be caused by direct interaction between the flagellum and pili that are known to be at the same pole of a *C. crescentus* swarmer cell. Such an interaction might be weaker and more transient at a lower viscosity, when the flagellum rotates with a higher speed. A faster rotating flagellum is expected to move past the surrounding pili faster, and perhaps less likely to get entangled, trapped, and stalled.

We observed no difference in the percentage of cells losing motility between the two Δpilin strains, with and without motor switching, in either the viscous or viscoelastic medium. This result suggests that the loss of motility, perhaps due to jamming or stalling of the motor, does not depend on motor switching mechanics. It also implies that, while increasing mechanical load on the flagellum can stall or jam the motor, the effect does not appear to be coupled to the switching behavior. Additional experiments were later designed to measure the motor switching behavior while varying the mechanical load, in order to further test this implication.

### Significant cell death occurred at high glycerol levels but not in PEG/PEO media of comparable viscosity

We performed a specific test to see whether the loss of motility at high viscosity was reversible using both Δpilin strains. We first mixed swarmer cells with 48% glycerol by mass, and noted that within minutes all cells stopped moving. We then diluted the mixture 1:1 with cell medium, so that the cells were eventually in 24% glycerol with a much reduced viscosity of 1.8 cP. Despite the expectation that over 60% cells would be motile based on Figure 3 for both strains if they were exposed to only 24% glycerol, all the cells that received a shock of 48% glycerol for a few minutes and then were reduced to 24% glycerol remained non-motile over a 30-minute observation period. We conclude that the loss of motility due to elevated levels of glycerol is irreversible.

**Figure 4:**
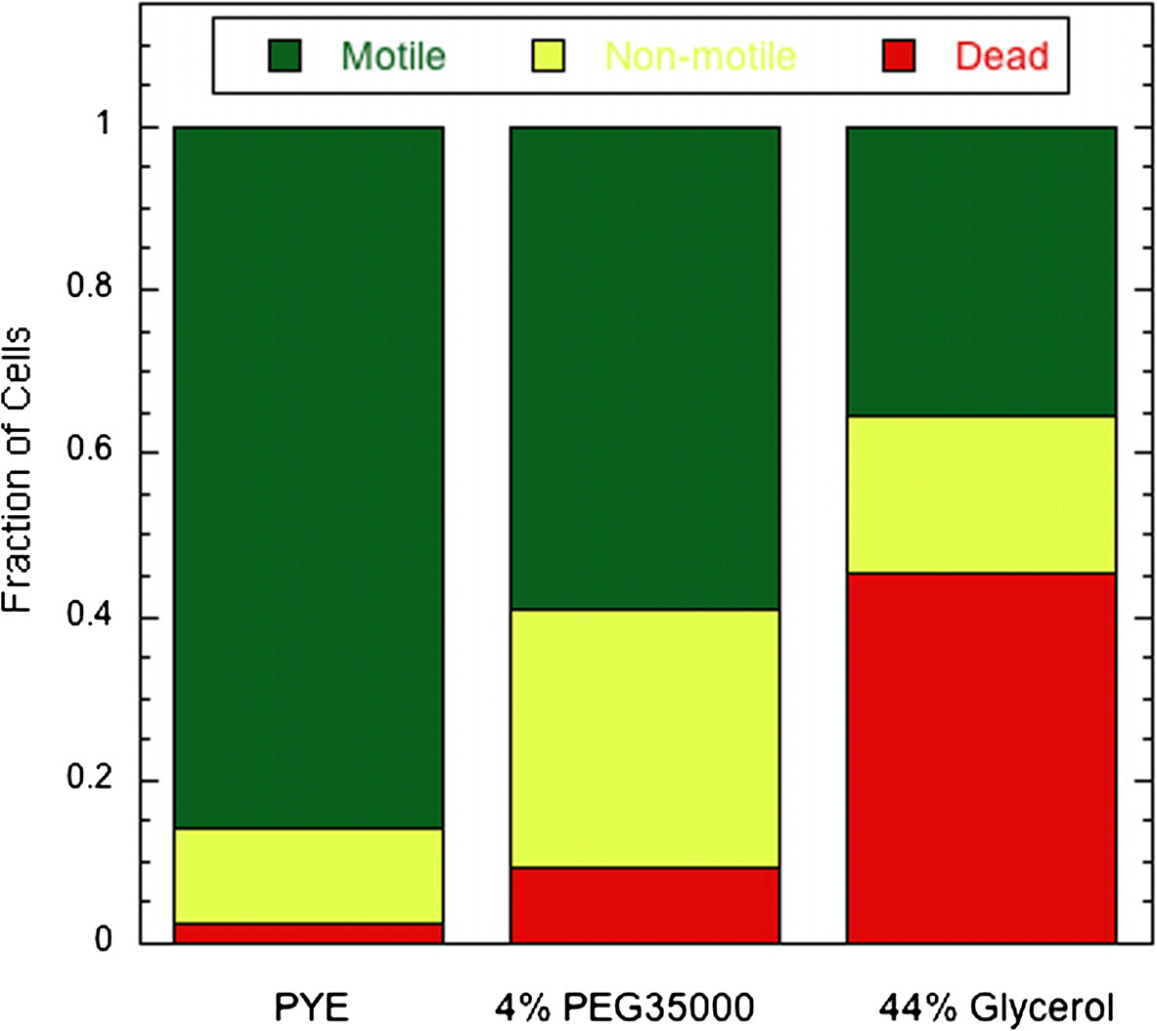
**Fractions of dead, non-motile and motile cells under three medium conditions.** Cells whose membrane became permeable to DiBAC were considered dead. The non-motile cells were those whose membrane was still intact, so that the cells were not labeled by DiBAC. Observations were made with SB3860 cells within minutes following the preparation as described under Materials and Methods. The fractions of motile cells were determined prior to the labeling experiments, with the results taken from Figure 3. The numbers of non-motile cells counted in the labeling experiments were 467 in PYE, 1207 in 4% PEG 35000, and 565 in 44% glycerol.

Fluorescence staining using DiBAC showed that a significant percentage of cells treated by 44% glycerol died within the minutes required to mix glycerol with the cells in the growth medium. Figure 4 shows that about 65% lost motility when mixed with 44% glycerol and over 70% of these non-motile cells became permeable to the dye and were practically dead. In contrast, among those cells found non-motile when mixed with 4% PEG 35000 (41% of total) or in PYE control (14% of total), only 23% and 18% of the non-motile cells were found dead, respectively, which might have occurred during the sample preparation such as the synchronization, mixing by pipetting, etc.

Previous work by others had shown that at an elevated level of ~50% or higher, glycerol caused the death of *E. coli*, due to severe osmotic stress or dehydration [36]. On the other hand, it is a widely accepted practice to store bacterial specimens at −80°C in relatively low concentrations of ~15-25% glycerol, in order to prevent ice crystal formation and rapid water loss during the freeze and thaw process [37]. Although we found no similar studies for *C. crescentus*, the findings for *E. coli* offers a crude guideline for the lethal level of glycerol [36,38], which appears consistent with the level we found for the total loss of motility and viability of *C. crescentus* (48%).

We also measured the osmolarity of 10% glycerol in comparison with that for 10% PEG 4000, 5% PEG 35000, and 5% PEO 400000. The results are listed in the Additional file 1: Table S1, confirming that glycerol produced much stronger osmotic pressure at comparable viscosity than that caused by introducing polymers into the cell medium. These additional measurements confirmed the mechanism detailed by earlier studies with glycerol, while also suggesting that adding inert polymers into the cell medium may be the better approach for the study of bacterial motility at elevated viscosity.

In conclusion, high percentage glycerol levels cause loss of motility and viability, likely attributable to hyperosmotic shock. In contrast, large PEG/PEO polymers appear to cause more moderate loss of motility by stalling the flagellar motor without killing the cells.

### Polymer matrix attenuates the decrease of swimming speed

Here we switch gears to describe our findings on the influences of glycerol and polymer matrices on the swimming speed of the cells that remain motile. A close comparison of these two types of media led to insights into the physical process. Implications of the features we observed are briefly discussed.

**Figure 5:**
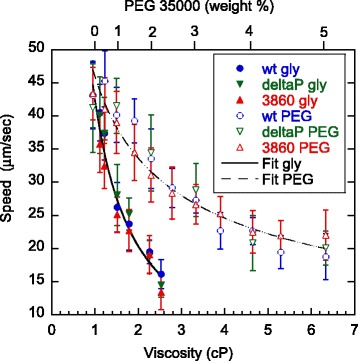
**Average swimming speed of cells as a function of viscosity, varied by addition of glycerol or PEG 35000.** The speed data were generated by MATLAB analysis of videos taken at 5 minutes after mixing bacteria with the viscous solution and within 15 minutes of synchronizing the swarmer cells. The solid lines are power law fits to collective data of all three strains, yielding exponents of −1.06 in viscous (glycerol) and −0.45 in viscoelastic (PEG 35000) solutions, respectively. The numbers of cells with their speeds measured were, for wild-type, 80 in PYE (control), 2084 in glycerol, and 773 in PEG 35000; for Δpilin, 23 in PYE, 270 in glycerol, and 170 in PEG 35000; and for SB3860, 181 in PYE, 1033 in glycerol, and 868 in PEG 35000. The error bars represent standard deviations, to convey the fact that there was a natural spread of swimming speed under each condition.

Based on previous reports, *C. crescentus* swarmer cells undergo “forward-backward-flick” motion as the flagellar motor switches between clockwise and counterclockwise rotations [39]. On average, the speed of the backward motion is the same as that of the forward motion in spite of subtle differences between their trajectories. As viscosity was increased, we observed decreasing swimming speeds of *C. crescentus* (Figure 5). The speeds of all three strains were very similar to each other in the viscoelastic solution containing PEG 35000 and the viscous, Newtonian solution containing glycerol. In glycerol, the speeds decreased inversely proportional to viscosity, with a fit power of −1.06 (R = 0.98). The inverse relationship suggests that the flagellar motor maintains a constant torque (proportional to speed times viscosity) as the viscosity of the medium varies. Indeed, the flagellar motor of *C. crescentus* has been previously shown to exert a constant torque at a range of rotation rates, from ~50 to ~300 revolutions per second, with the upper bound being the motor speed found during free swimming in water [15]. The new finding confirms that, at the high load regime, the flagellar motor maintains a constant torque regardless of its rotational rate, which decreases as the viscosity of the surrounding medium is increased [14,15,40]. The motor rotation is known to involve a flux of protons through the MotA/MotB stator complex [1,41,42]. Our finding of constant torque also implies that the viscous agent, including even the fairly small glycerol molecules, does not interfere with the proton-motive path of the MotA/MotB complexes, which would otherwise have impeded the proton flow and altered the motor torque in some dose-dependent fashion. Note that our study is conducted under the high load regime in that all MotA/MotB units are presumably coupled with the flagellum motor. This scenario is rather different from the low motor load regime, under which interesting findings have been made recently on the flagellar motor of *E. coli*, which adapts to an increasing load with stepwise deployment of additional units, thereby increasing torque [2,43-45].

In PEG 35000, we found a more gradual decrease in swimming speed with increasing viscosity (fit power −0.45; R = 0.96). In other words, the polymer network of PEG allowed for markedly higher speed as compared to an equally viscous solution of glycerol. This experimental result suggests that the swimming speed driven by a rotating flagellum is enhanced by the elasticity of the polymer matrix. In fact, more pronounced enhancements on swimming by a viscoelastic medium were previously shown on several other species of bacteria [6-8]. Note those earlier works used methylcellulose and polyvinylpyrrolidone (PVP), which are much larger in size than the polymers we used in this study. When we performed similar experiments with *C. crescentus* using methylcellulose, we observed a peak in swimming speed at ~0.05% (viscosity ~1.4 cP; data not shown). The latter result is consistent with previous observations of other species of bacteria [6-8]. The comparison here suggests that the difference in speed-viscosity behavior between our measured speeds in PEG 35000 (no peak) and those previously published by others using methylcellulose and PVP is likely due to the difference in matrix molecular size rather than species-specificity. The extremely large sizes of methylcellulose and PVP (on the order of a million daltons) appear to have caused enough enhancements on thrust, over-compensating the expected decrease in swimming speed due to increased drag. As a result, a peak in swimming speed has been predicted based on hydrodynamic calculations [11,46]. Unfortunately, a rigorous comparison between the theoretical predictions and the experimental results is hampered by not knowing the actual size of the extremely high molecular weight polymers used in those experiments, and at the present time a lack of rheology data for those polymers at the low concentrations, which are necessary for bacteria to remain motile. In fact, our ongoing effort is designed to be conducive to more rigorous mechanical analysis using PEG and PEO, which cover a large range of available sizes.

### Motor switching and stalling may be differentially regulated

**Figure 6:**
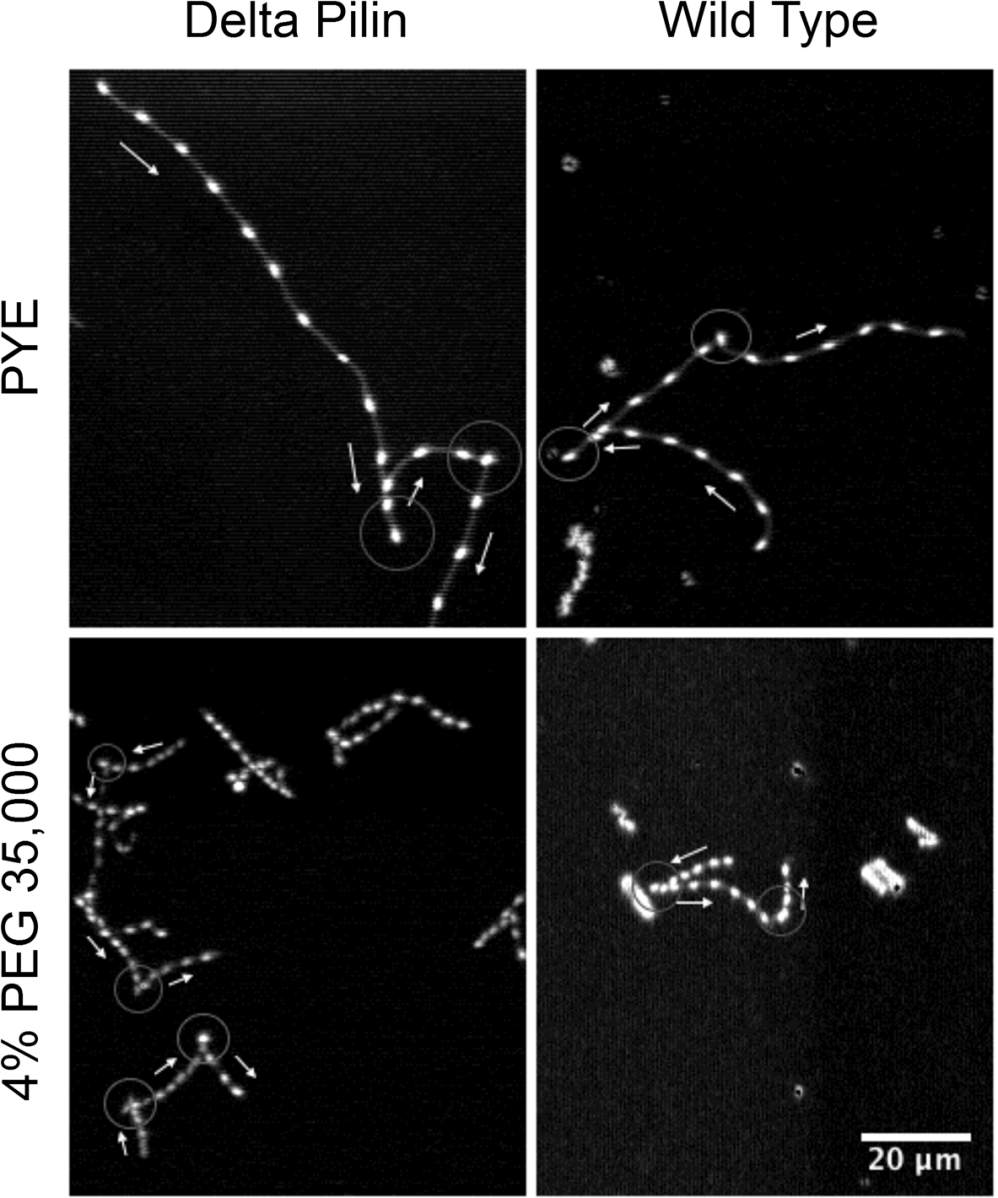
**Overlays of selected frames at 0.17 s intervals out of a 3-second movie, to illustrate switches during the motion of** Δ**pilin (left panels) and wild-type (right panels) cells in PYE (top panels) and 4% PEG 35000 (bottom panels).** The faint grey lines following the swimming trajectories were produced with darkened overlay of all frames over the 3-second movies at 125 frames/second. Arrows next to the trajectories were drawn to point out the directions of motion, with switches indicated by grey circles. The scale bar applies to all 4 images.

We further explored whether a viscoelastic medium might affect the motor switching behavior by increasing the mechanical load on the flagellum. We measured the average switching frequency of wild-type and Δpilin cells in the 4% PEG medium in comparison with that of PYE. A set of four movie clips via links in the Additional files 2, 3, 4 and 5 illustrates switching events that occurred for both wild-type and Δpilin cells in PYE and 4% PEG 35000 media. Figure 6 shows overlays of selected movie frames where cells appear as bright spots and locations of switches are marked. The high frame rate of the recorded movies ensured sufficiently high time resolution of ~10 ms for our measurements (See details in Materials and Methods).

**Figure 7:**
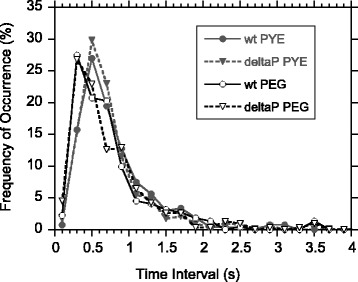
**Histograms of intervals between motor switches.** The intervals were measured for wild-type (wt, circles) and Δpilin (deltaP, triangles) cells in the standard PYE medium (solid symbols) and in the PYE medium containing 4% PEG 35000 (hollow symbols). The data show slight shift of switch intervals to lower values due to addition of PEG 35000. More details of these data are tabulated in Table 2.

A close comparison of switching behavior of both wild-type and Δpilin strains was made, with results shown in Figure 7 and Table 2. We found no significant difference between the cells with and without pili (Figure 7). There is a small increase in switching frequency for both cell types in 4% PEG 35000 medium, but the visual displacement in peak position is much smaller than the natural spread of the measured intervals. A Student *T*-test performed in comparison with the average intervals confirms no significant difference. Note also the large standard deviations of the switching intervals under all 4 conditions (Table 2). The simplest yet an important conclusion of this comparison is that there was insignificant difference among all 4 measured conditions. It was not possible to perform the same measurements for cells in glycerol-PYE medium of comparable viscosity to that of 4% PEG (4.6 cP). This would have required 48% glycerol, at which concentration all cells totally lost their motility.

**Table 2:** **Comparison in average intervals between switches of motor rotation**

	**Wild-type in PYE**	**Wild-type in PEG 35000**	Δ**pilin in PYE**	Δ**pilin in PEG 35000**
Number of Switching Intervals	267	222	527	310
Average Interval (s)	0.81	0.74	0.76	0.73
Standard Error (s)	0.033	0.037	0.024	0.033
Standard Deviation (s)	0.52	0.55	0.55	0.57
Median (s)	0.64	0.59	0.61	0.57

Comparison of average switch intervals with standard errors, standard deviations, and median intervals, based on the number of measured intervals listed for wild-type and Δpilin cells in PYE with and without 4% PEG35000. Note the large values of the standard deviations due to the natural spread of switch intervals. Nevertheless, the average interval is nearly the same under all 4 conditions.

Our findings from the switching interval measurements strongly suggest that the switching and stalling of the flagellar motor are differentially regulated. The gradual loss of motility in either a viscous or viscoelastic medium does not depend on whether or not the motor switches (Figure 2); the motor switching frequency varies little with the addition of PEG 35000 up to 4%, which increases the apparent viscosity 5 fold. The loss of motility appears to be a sudden stall or jam, either due to injury caused by hyperosmotic shock at elevated levels of glycerol, or triggered by some mechanical failure under a high mechanical load or stress. Once the stalling occurs, the damage to the motor appears permanent, evidenced by the irrecoverable loss of motor function, and subsequent loss of the cell’s viability. We note that under the natural environment of relatively low fluid viscosity, the *C. crescentus* flagellar motor frequently switches its rotation direction with very short or even non-detectable stall time. In contrast, there are also species such as *R. sphaeroides* for which the motor can pause as part of its natural cycle of motion [47]. We observed a few instances when a *Caulobacter* cell paused and then resumed motility.

## Conclusions

This study was designed to assess and differentiate effects of solution viscosity and viscoelasticity on the swimming behavior of *C. crescentus* as a simple micro-swimmer with one helical flagellum. The results clearly show quantitative differences between a polymer matrix as a viscoelastic medium and a Newtonian fluid with elevated viscosity. In the Newtonian fluid, we found the swimming speed to scale inversely proportional to viscosity, as expected based on hydrodynamic theory for constant motor torque. In the test polymer network, we found the swimming speed to fall more gradually with viscosity, as v ~ η^-0.45^. This result suggests that the flagellated micro-swimmer produces a larger thrust at an increased polymer concentration, which partially offsets increased drag on the cell body. We were unable to quantitatively account for the measured results based on fluid mechanical calculations, due to lack of knowledge of detailed rheological properties of the PEG medium. Specifically, the apparent viscosity measurements performed in this study do not yield values of the storage and loss moduli as functions of the frequency of perturbance exerted by the rotating flagellum. Thus, additional characterization of the polymer matrix is required to better define the mechanical effects of the chosen media on the bacterial swimming speed.

By performing experiments using three selected strains, we were able to distinguish common behavior robustly accounted for by mechanical properties of the media with some notable exceptions, which offer insights into the functions of the flagella motor. The quantitative dependence of swimming speed on viscosity or concentration of polymers added is practically identical among all three strains. In both media, there is a significant difference in fraction of immobilized cells observed between the wild-type cells and the two strains lacking pili. Additionally, we saw nearly no effect of a viscoelastic medium on the switching frequency of the flagellar motor of *C. crescentus*. The results in this report call for further study on the control of the flagellar motor, as well as more detailed mechanical analysis. The latter requires high-speed imaging of the flagellum in order to determine its relative orientation to the cell body, as well as the hook that connects them. On the other hand, a more comprehensive characterization of the frequency dependent rheological properties of the polymer matrix is also required at the range most relevant to bacterial motility.

We conclude by suggesting that our study of bacterial motility in complex fluids using *C. crescentus* as a model system may spark interest in similar experiments on other flagellated bacteria in diverse environments, particularly those that find viscoelastic media their natural habitat.

## Methods

### Bacterial strains

*Caulobacter crescentus* strains CB15 wt, CB15 Δpilin (YB375) [33], and SB3860 were used. SB3860 is cheR138::Tn5 in YB375, ie., a mutant of CB15 Δpilin. It was kindly generated by Bert Ely, and was used in our previous studies [34,35]. As described previously [33,48], the strains were grown in peptone yeast extract (PYE) medium [23] and synchronized with the plate release method [49], modified recently [22,48]. The synchronization procedure yielded swarmer cells in fresh PYE that were within 5 minutes of division. The synchronized swarmer cells were immediately used in the experiments. Video recordings were terminated before the swarmer cells reached the age of 30 minutes, ensuring that properties observed were not complicated by the onset of differentiation towards the non-motile stage.

### Viscometry and osmolarity measurements

We used polyethylene glycol of average sizes of 35,000 and 4000 daltons (PEG 35000 and PEG 4000, respectively; Sigma-Aldrich) and polyethylene oxide of 400,000 daltons (PEO 400000, Sigma-Aldrich). PEG 35000 has a radius of gyration of 8.6 nm [16,50], and the polymer becomes entangled in solutions at about 2% by weight or mass, estimated based on the Rouse model of polymer chains [51,52]. The corresponding numbers for PEG 4000 are 2.9 nm and 6% and for PEO 400000 are 29 nm and 0.6%, respectively. Solutions of PEG 35000 or glycerol (Fisher Scientific) in PYE medium at room temperature were prepared to vary their viscosity up to 5–7 times to water. The PEG(PEO)/PYE and glycerol/PYE mixtures were made by first weighing a highly viscous stock solution such as 40% PEG 35000 (in PYE), 5% PEO 400000 (in PYE), or 100% glycerol, using an analytical balance, and then diluting it with the desired amount of PYE. The concentrations reported are by mass or weight, also commonly referred to as wt/wt percentage. Smaller sets of measurements were performed using methylcellulose (Sigma-Aldrich), which was prepared similarly by diluting from a 1% stock solution. The shear viscosities of the mixtures were measured using a size 50 pre-calibrated Cannon-Fenske Routine viscometer (CFRC 9721-B50 series, CANNON® Instrument Company, State College, PA). The measured viscosity values for PYE medium showed no significant difference from that of pure water. The measured values for glycerol-PYE mixtures were found in excellent agreement with those listed for glycerol-water mixtures in the Chemical Rubber Company (CRC) handbook with a temperature-related correction, as shown in the Additional file 1. Specifically, our viscosity measurements were performed at a room temperature of ~24°C, yielding values about 10% lower than those listed in CRC at 20°C.

We used the calibrated viscometer to measure the shear viscosity of the cell medium containing PEG or PEO. Solutions containing PEG 4000 over the 0-10% range yielded viscosities up to 3.0 cP. Solutions containing PEG 35000 over the 0-5% range yielded viscosities up to 6.5 cP. Solutions containing PEO 400000 over the 0-1% range yielded viscosities up to ~15 cP. We searched the literature and found these values to be consistent with values measured for PEG of several other molecular sizes, considering the known trend of a sharp increase with molecular weight at fixed mass concentration [50]. Our measured values are tabulated and plotted in Additional file 1.

We measured the osmolarity of 10% glycerol, the PYE growth medium and selected PEG/PEO solutions, using a commercial osmometer based on freeze point depression detection (Osmette II, Precision Systems, Model 5005, Natick, MA).

### Measurements of motility

An aliquot of synchronized cells in PYE was mixed with a viscous medium in a 1 mL centrifuge tube, by gently pipetting for 2–5 minutes, to uniformity. The final mixtures contained up to 10% of PEG 4000, 5% of PEG 35000, 0.5% of PEO 400000, or 48% of glycerol, all by mass. A 115–150 μL aliquot of bacteria-viscous agent mixture was then placed within a chamber formed by a 12.7 mm-diameter rubber O-ring, glued on top of a microscope slide using epoxy. The bacteria sample was made with sufficient depth (~1 mm) in order to collect data about swimming behavior far from the fluid boundary. A cover slip was placed on top of the O-ring filled with the sample to form a seal and allow for convenient observation using an upright microscope. Movies for counting motile versus non-motile cells were recorded between 2 and 5 minutes after mixing unless longer times were specified.

Images and videos were taken using a Nikon Eclipse E800 upright optical microscope with a mounted CoolSnap CCD camera (Photometrics, Tucson, AZ) and the software MetaMorph (Universal Imaging Corp., 2002). In order to capture *C. crescentus* during the swarmer stage of its life cycle, all observations were made on swarmer cells within 30 minutes in age.

To collect videos for motility detection, we used a long working distance 40x phase-contrast objective with a 0.05 s exposure time and a low capture rate of 6.7 frames/second. Under the 40x objective magnification, individual swarmer cells were discernable as opposed to cell aggregates or pre-divisional cells, which were both excluded from the counts. In contrast to swimming cells, which moved several microns per frame, non-motile cells displayed only diffusive motion and moved much less than 1 μm/s. Since the difference between them was over tenfold, the motile and non-motile cells were distinguished by eye. Percentages of immobilized cells were obtained by counting single swarmer cells that did not swim compared with those that were swimming in the recorded short videos.

Two methods were used to yield appropriate error bars. Method one applies to the percent motile data for strain 3860 in PEG 4000 and PEO 400000. Ten videos of ten frames each were taken within two minutes. Within each video, a few dozen cells were counted, out of which one value of percent motile was obtained. These ten values were averaged to yield the percentage motile along with an error bar. Method two applies to the percent motile data of all three strains in glycerol and PEG 35000. It extends beyond method one as similar measurements under these conditions were repeated two more rounds. The final percent motile data were shown as the average from the three rounds of measurements and the standard error from the three values. The errors are smaller by the second method since three times as many cells were counted under each condition of those measurements.

### Measurements of swimming speed

For measurements of swimming speed, samples were prepared using the same rubber o-ring as for the motility assay. A sample was typically placed on the microscope stage for 5–7 minutes after mixing before recording to ensure the decay of fluid flow, which was the main source of error. A 20x objective was used with the dark-field setting of the microscope to take videos with 0.1s exposure time at 6.7 frames/second for 20–50 frames. The swimming speeds were obtained by analyzing the dark field videos using a custom-written MATLAB (Mathworks, Inc., Natick, MA) program. The program selects for swimming trajectories with positions discernable over ≥10 frames and without sharp changes of direction (threshold set at 1 radian/second) between frames in order to exclude measuring speed during motor switching events. Instantaneous speed was determined based on the spacing between consecutive cell body positions. The average speed over a short trajectory of each cell (during either forward or backward motion, as the difference in speed is negligible) was calculated for dozens or hundreds of cells under each condition. Due to the natural spread of swimming speed among individual cells, our data were plotted with standard deviation, which does not depend on how many cells were measured under each condition.

### Measurements of cell viability

Following an established method [53], we used a fluorescent dye called DiBAC(4)3, (bis-[1,3-dibutylbarbiturate] trimethine oxonol) (Sigma-Aldrich, Inc.), which enters depolarized cells and exhibits enhanced fluorescence. Thus the DiBAC dye was chosen to label those *C. crescentus* cells that had lost their membrane integrity and were considered dead. A stock solution of 1 mg/mL DiBAC was dissolved in 1:1 ethanol:water mixture and diluted 1:1000 into synchronized swarmer cells in PYE (control), PYE containing 44% glycerol, or PYE containing 4% PEG 35000. Thin samples were prepared the same way as described below for the motor switching experiment. Each sample was imaged using both phase contrast and fluorescence microscopy. A long exposure time (0.5 s) was used so that motile cells appeared as faint streaks under phase contrast. The number of non-motile cells appearing in each image was counted and the fraction of non-motile cells that were dead (i.e. appeared under both phase contrast and fluorescence) was calculated.

### Measurements of motor switching

For measurements of motor switching, thinner samples were found to yield better images as the cells were prevented from moving out of the focal plane. A 5 μL sample was pressed between a microscope slide and a coverslip, sealed with a layer of vacuum grease around the edges. The sample thickness of such preparations was on the order of 10 μm.

Videos were taken using a Photron FASTCAM-PCI R2 high-speed camera mounted to a Nikon Eclipse TE2000-U inverted optical microscope. Videos were recorded using a 20x phase-contrast objective with a frame rate of 125 frames/second. The time interval between motor switches was measured by visually identifying the frames at which a particular cell underwent a reversal in its direction of travel. The time interval between two consecutive switching events was then calculated based on the known video frame rate. The high frame rate ensured that the measurement error was within 10 milliseconds, far shorter than the intervals between motor switches, which typically occur on the order of a second.

## Additional files

Additional file 1:
**Additional information on viscosity and osmolarity.**
Additional file 2:
**Movie 1, showing wild-type cells moving in PYE medium.**
Additional file 3:
**Movie 2, showing delta pilin cells moving in PYE medium.**
Additional file 4:
**Movie 3, showing wild-type cells moving in PEG 35000.**
Additional file 5:
**Movie 4, showing delta pilin cells moving in PEG 35000.**
