# Supplementary material for: Altered motility of Caulobacter Crescentus in viscous and viscoelastic media
Source: BMC Microbiol. 2014 Dec 24;14:322. doi: 10.1186/s12866-014-0322-3 (PMC4302598; doi:10.1186/s12866-014-0322-3)
Supplement: Additional file 1: — Additional information on viscosity and osmolarity. [file 12866_2014_322_MOESM1_ESM.pdf]

Altered Motility of *Caulobacter Crescentus* in Viscous and Viscoelastic Media

Yukun Gao, Marianna Neubauer, Alexander Yang, Nathan Johnson, Michael Morse, Guanglai Li, and Jay X. Tang (Physics Department, Brown University, Providence, RI 02912)

Additional Information

I. Viscometer Calibration and Temperature Correction

As a form of calibration, we used a commercial Cannon-Fenske Routine viscometer to measure the viscosity of glycerol-water mixtures up to 50% by weight. All our viscosity measurements and microscopy study were performed at room temperature of ~24°C. The viscosity of aqueous glycerol solutions is known to be a highly sensitive function of temperature, showing 20-30% drops from 20°C to 30°C [1, 2]. Either a linear interpolation, or a more comprehensive treatment as published recently by Cheng [3], accounts for on average about 10% drop from the Chemical Rubber Company (CRC) listed values at 20°C to expected values at 24°C. We saw a good agreement between our measured values performed in three trials and the CRC values, factored by a 10% reduction (Figure A1). This good agreement assures us that the commercial viscometer was indeed well calibrated, at least in the viscosity range of this study.

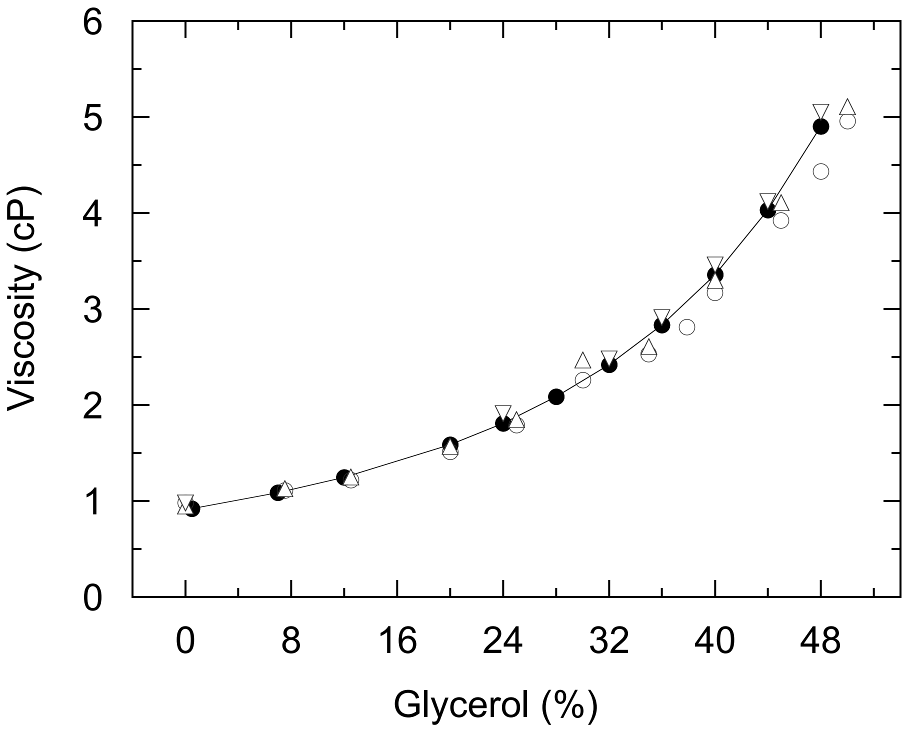

Figure A1. Measured values of viscosity of glycerol-cell medium mixtures in comparison with CRC listed values for glycerol-water mixtures. Hollowed symbols represent three rounds of measurements at room temperature. Solid circles connected by a solid line are values scaled down by 10% from the CRC table at 20°C, to correct for temperature dependence of viscosity. The excellent agreement confirms that the viscosity values measured in this work were reliably calibrated.

II. Viscosity of PEG 4000, PEG 35000, and PEO 400000 solutions at selected polymer concentrations

We used solutions of long, linear, and inert polyethylene glycol of average molecular weights of 35000 daltons (PEG 35000) and 4000 daltons (PEG 4000), as well as even longer polymer of polyethylene oxide of 400000 daltons (PEO400000), in order to explore the interaction of motile bacteria with a viscoelastic medium. Over the past century, there has been extensive study on the rheological properties of polymers in good solvents, such as PEG or PEO in water. Due to numerous types of polymers and variable degree of polymerization available among commercial products, however, values of the particular

solutions of PEG 35000 used extensively in this study were not found in the literature. Therefore, we measured the shear viscosity of PEG 35000 of a number of concentrations covering the range of 0-5% by weight, in comparison to that of PEG 4000 in the range of 1-10% by weight, as well as PEO400000 up to 1% only. The measured values are listed in Table 1 in the result section of the paper. Our measured values are also in line with published values for PEG of comparable sizes, such as PEG 6000-20000 by Holyst *et al.* [4], noting that at a fixed weight percentage the longer the polymer, the more viscous the solution is.

Since the majority of our study was performed using PEG 35000, we sought an analytical fit in the viscosity-polymer concentration dependence. Previous work, such as the 2009 Holyst paper [4], focuses on measurements performed in solutions of PEG beyond overlap concentration. Under those conditions, high molecular weight PEG polymers form an entangled network, giving rise to not only high shear viscosity, but also viscoelastic behavior. The range of PEG concentrations used in our study, however, falls on the lower end of those modeled in the Holyst paper. The overlap concentration is ~2.1% for PEG 35000, estimated by using it radius of gyration R_g=8.6 nm as the size of the polymer [4, 5]. We found the stretched exponential form derived from the master curve there does not fit our measured data. Instead, we applied a simpler quadratic power law fit to yield an analytical relation between viscosity and percentage of PEG, which yields $\eta=1.01+0.36*[\%PEG]+0.14*[\%PEG]^2$ (Figure A2). We note here the empirical nature of both fit forms, and the fact that there is to our knowledge no sound analytical relation known in the literature for the dynamic viscosity of linear polymers in weak entanglement regime, which is intermediate between dilute and high entanglement limits. A survey of viscoelastic properties of various polymer solutions goes beyond the scope of this study. Nevertheless, the common occurrence of polymer solutions of low and moderate levels of viscosity in our view deserves more extensive study in the future.

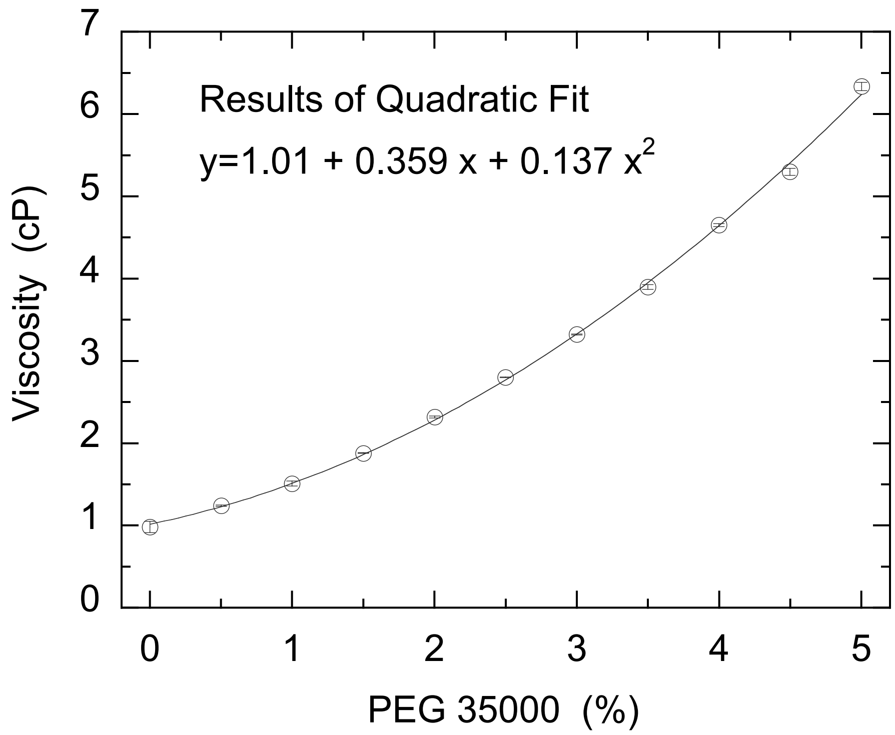

Figure A2. Measured viscosity of PEG 35000 as a function of the polymer weight percentage. The data were fit empirically to a quadratic function, with obtained parameters indicated on the graph.

III. Osmolarity of PEG 4000, PEG 35000 and PEO 400000 solutions at selected concentrations

We measured the increase of osmolarity due to addition of PEG or PEO into the motility buffer in order to assess whether altered motility might be attributable to such changes. The results shown below in Table A1 are consistent with those reported in the literature, showing orders of magnitude stronger osmotic effect of glycerol than the upper limit of concentrations of all the polymer solutions used, such as 5% by mass.

Solution	PYE	PEG 4000 (5%)	PEG 35000 (5%)	PEO 400000 (5%)	Glycerol (10%)
Osmolarity (mOsm)	20.7	35.0	23.5	26.0	1251

Table A1. Measured osmolarity of PEG 4000, PEG 35000 and PEO 400000 in water, in comparison with 10% glycerol and the cell medium (PYE). Each value is an average of two or three measurements, which varied by no more than 10%.

IV. Additional References

1. Segur, J.B. and H.E. Oberstar, *Viscosity of Glycerol and Its Aqueous Solutions,* Industrial and Engineering Chemistry, 1951. **43**(9): p. 2117-2120.
2. Sheely, M.L., *Glycerol Viscosity Tables,* Industrial and Engineering Chemistry, 1932. **24**(9): p. 1060-1064.
3. Cheng, N.-S., *Formula for the Viscosity of a Glycerol-Water Mixture,* Industrial and Engineering Chemistry Research, 2008. **47**: p. 3285-3288.
4. Holyst, R., A. Bielejewska, J. Szymanski, A. Wilk, A. Patkowski, J. Gapinski, A. Zywocinski, T. Kalwarczyk, E. Kalwarczyk, M. Tabaka, N. Ziebacz, and S.A. Wieczorek, *Scaling Form of Viscosity at All Length-Scales in Poly(Ethylene Glycol) Solutions Studied by Fluorescence Correlation Spectroscopy and Capillary Electrophoresis,* Physical Chemistry and Chemical Physics: PCCP, 2009. **11**(40): p. 9025-9032.
5. Ferry, J., *Viscoelastic Properties of Polymers*. 3rd ed. 1980, New York: John Wiley.
